# A Novel Role of the NRF2 Transcription Factor in the Regulation of Arsenite-Mediated Keratin 16 Gene Expression in Human Keratinocytes

**DOI:** 10.1289/ehp.10696

**Published:** 2008-03-12

**Authors:** Hitoshi Endo, Yoshihiko Sugioka, Yoshihiko Nakagi, Yasuaki Saijo, Takahiko Yoshida

**Affiliations:** Department of Health Science, Asahikawa Medical College, Midorigaoka, Asahikawa, Hokkaido, Japan

**Keywords:** antioxidant response element, arsenic, hyperkeratosis, keratin 16 (*K16*), keratinocytes, nuclear factor erythroid-derived 2 related factor 2 (NRF2), transcription

## Abstract

**Background:**

Inorganic sodium arsenite (iAs) is a ubiquitous environmental contaminant and is associated with an increased risk of skin hyperkeratosis and cancer.

**Objectives:**

We investigated the molecular mechanisms underlying the regulation of the keratin 16 (*K16*) gene by iAs in the human keratinocyte cell line HaCaT.

**Methods:**

We performed reverse transcriptase polymerase chain reaction, luciferase assays, Western blots, and electrophoretic mobility shift assays to determine the transcriptional regulation of the *K16* gene by iAs. We used gene overexpression approaches to elucidate the nuclear factor erythroid-derived 2 related factor 2 (NRF2) involved in the *K16* induction.

**Results:**

iAs induced the mRNA and protein expression of *K16*. We also found that the expression of *K16* was transcriptionally induced by iAs through activator protein-1–like sites and an antioxidant response element (ARE) in its gene promoter region. Treatment with iAs also enhanced the production and translocation of the NRF2 transcription factor, an ARE-binding protein, into the nucleus without modification of its mRNA expression. In addition, iAs elongated the half-life of the NRF2 protein. When overexpressed in HaCaT cells, NRF2 was also directly involved in not only the up-regulation of the detoxification gene thioredoxin but also *K16* gene expression.

**Conclusions:**

Our data clearly indicate that the *K16* gene is a novel target of NRF2. Furthermore, our findings also suggest that NRF2 has opposing roles in the cell—in the activation of detoxification pathways and in promoting the development of skin disorders.

Inorganic sodium arsenite (iAs), a ubiquitous element, is one of the most toxic metals present in the natural environment (Bagla and Kaiser 1996). Arsenicals are found as naturally occurring constituents of soil, food, and drinking water (Wu et al. 1989; Yoshida et al. 2004), and exposure to iAs has been associated with a variety of disease outcomes, including disorders of the skin, urinary bladder, liver, and lung (Tchounwou et al. 2004). In particular, skin hyperkeratosis is a characteristic dermatologic lesion associated with ingestion of arsenic from contaminated groundwater (McLellan 2002; Yoshida et al. 2004). There is also a significant association between hyperkeratosis, nonmelanoma skin cancer (e.g., basal cell carcinoma and squamous cell carcinoma), and Bowen disease (Col et al. 1999; Rossman et al. 2004). Furthermore, the pathologic features associated with arsenic-induced hyperkeratosis present as typical acanthotic types of psoriasis-like keratosis, characterized by the aberrant proliferation and terminal differentiation of epidermal keratinocytes (Lee et al. 2006). Many epidemiologic studies have shown that hyperkeratoses are the most frequent precursor lesions of some skin cancers (Bagla and Kaiser 1996; Col et al. 1999).

The keratins are the most prominent cytoskeletal proteins in keratinocytes and comprise a large family of proteins that form intermediate filament networks in all epithelial cell types (Moll et al. 1982). Keratin 16 (*K16* ) and keratin 6 *(K6*) genes are constitutively expressed in a number of stratified epithelial levels, including the palmar and plantar epidermis (Moll et al. 1982). In skin diseases characterized by aberrant proliferation and differentiation, such as psoriasis and cancer, K16 is detectable at higher levels compared with normal tissue (Haider et al. 2006). Furthermore, the tissue-specific overexpression of wild-type *K16* in the epidermis of transgenic mice results in the hyperproliferation of keratinocytes and aberrant keratinization of cornified layers, leading to hyperkeratosis of the skin (Takahashi et al. 1994).

Nuclear factor erythoid-derived 2 related factor 2 (NRF2), a “cap ’n’ collar” basic leucine zipper transcription factor, regulates a transcriptional program that maintains cellular redox homeostasis and protects cells from oxidative stress and xenobiotic agents (Ishii et al. 2000; Moi et al. 1994). Several detoxifying and antioxidant genes, including glutathione-*S*-transferases (*GSTs*), heme oxygenase-1 (*HMOX1*), and thioredoxin (*TXN*), are regulated by NRF2 through the antioxidant responsive element (ARE) in the respective promoter regions of these genes (McMahon et al. 2001; Wakabayashi et al. 2004). NRF2 is held in the cytoplasm by a cytoskeletal-associated specific inhibitory protein (kelch-like ECH-associated protein 1; KEAP1) under normal cellular redox state conditions, where it is continuously targeted by the proteasomal degradation pathway (McMahon et al. 2003). Upon exposure of the cell to oxidative stress or electrophiles, NRF2 can escape this KEAP1-mediated repression, translocate to the nucleus, and activate the expression of its target genes (Dinkova-Kostova et al. 2002; McMahon et al. 2003).

Recently, studies of *Keap1*^–^/^–^mice have shown that NRF2 accumulates in the nucleus and constitutively activates the transcription of its target genes, even in the absence of stress signals (Wakabayashi et al. 2003). Most interestingly, however, the skin, esophagus, and forestomach of *Keap1*-deficient mice show cornified layer and hyperkeratosis phenotypes. In addition, previous studies have also shown that the expression of NRF2 and ARE-controlled genes is induced by iAs in some cell types (Pi et al. 2003; Sakurai et al. 2005). Furthermore, histochemical analyses have indicated that the expression of *K16* is increased in Bowen disease, basal cell carcinoma, and squamous cell carcinoma induced by arsenicals (Yu et al. 1993). However, it remains to be determined whether NRF2 can regulate the transcriptional activation of *K16* upon iAs exposure in human keratinocytes. Hence, these findings prompted us to investigate the molecular mechanisms underlying the regulation of the *K16* gene by iAs-induced NRF2 mediation.

## Materials and Methods

### Chemicals and reagents

A purified preparation of inorganic sodium arsenite (iAs; NaAsO_2_; Merck, Darmstadt, Germany) was dissolved in phosphate-buffered saline (PBS) and added directly to the culture medium. A fresh iAs solution was prepared for each new experiment. Cycloheximide (CHX), dimethyl-sulfoxide (DMSO), and a protease inhibitor cocktail were purchased from Sigma (St. Louis, MO, USA). CHX was dissolved in DMSO and stored –20°C until use.

### Cells and culture conditions

The human keratinocyte HaCaT cell line was obtained from N.E. Fusenig (German Cancer Research Center, Heidelberg, Germany). Cells were maintained in monolayer cultures in 95% air and 5% CO_2_ at 37°C in Dulbecco’s modified Eagles medium (DMEM) supplemented with 10% fetal bovine serum (FBS), 50 U/mL penicillin and 50 mg/mL streptomycin and nonessential amino acids (Gibco BRL, Paisley, UK).

### RNA preparation and semiquantitative reverse transcriptase-polymerase chain reaction (RT-PCR) analysis

We determined RNA expression levels by semiquantitative RT-PCR analysis as described previously (Sugioka et al. 2004). Total RNA was isolated from HaCaT cells using the GeneElute Mammalian Total RNA Kit (Sigma). The specific primers used for first-strand cDNA synthesis and PCR were as follows: *K16* [forward, 5′-GAT GCT TGC TCT GAG AGG TC-3′, and reverse, 5′-CCA GCA AGA TCT GGT ACT CC-3′; Gene Bank accession no. NM_005557 (National Center for Biotechnology Information 2007)]; c-*Jun* (forward, 5′-CCT GTT GCG GCC CCG AAA CT-3′, and reverse, 5′-ACC ATG CCT GCC CCG TTG AC-3′; NM_002228); *c*-*Fos* (forward, 5′-TTT GCC TAA CCG CCA CGA TGA T-3′, and reverse, 5′-TTG CCG CTT TCT GCC ACC TC-3′; NM_005252); *NRF2* (forward, 5′-AGA TTC ACA GGC CTT TCT CG-3′, and reverse, 5′-CAG CTC TCC CTA CCG TTG GA-3′; AF323119); *KEAP1* (forward, 5′-CAG AGG TGG TGG TGT TGC TTA T-3′, and reverse, 5′-AGC TCG TTC ATG ATG CCA AAG-3′; NM_012289); *TXN* (forward, 5′-CAG GGG AAT GAA AGA AAG G-3′, and reverse, 5′-CAA GGT GAA GCA GAT CG-3′; NM_003329), and glyceraldehyde 3-phosphate dehydrogenase (*GAPDH*) as a loading control (forward, 5′-ACC ACA GTC CAT GCC ATC AC-3′, and reverse, 5′-TCC ACC ACC CTG TTG CTG TA-3′, NM_002046). PCR products were separated on a 1.8% agarose gel and stained with ethidium bromide.

### Western blot analysis

We performed Western blot analysis as described previously (Sugioka et al. 2004). Briefly, nuclear and cytoplasmic proteins were extracted using the NE-PER nuclear and cytoplasmic extraction kit (Pierce, Rockford, IL, USA) according to the manufacturer’s protocol. For protein extraction, the cells were lysed in a buffer containing complete protease inhibitor cocktail. We measured protein concentrations using the DC Protein Assay Kit (Bio-Rad, Richmond, CA, USA). Equal amounts of protein were then resolved by sodium dodecyl sulfate-polyacrylamide gel electrophoresis (SDS-PAGE) and transferred to a polyvinylidene fluoride membrane (Amersham Biosciences, Bucks, UK). Immunoblotting was carried out with specific antibodies in Tris-buffered saline with 0.05% Tween 20. The primary antibodies were as follows: K16 (Neomarkers, Fremont, CA, USA), NRF2 and KEAP1 (Santa Cruz Biotechnology, Santa Cruz, CA, USA), c-Jun (Cell Signaling, Beverly, MA, USA), and β-actin (Sigma). After washing, the membranes were probed with horseradish peroxidase-conjugated secondary antibodies and developed by chemiluminescence using the ECL Plus Detection Kit (Amersham Biosciences).

### Plasmids, transfections, and luciferase assays

Human *K16* promoter regions of varying lengths (pXK-1, 3, 4, 5-1, and 5-2) were provided by Y-N Wang (National Cheng Kung University, Taiwan). These DNA fragments were prepared from HaCaT cells and were ligated into the pXP-1 luciferase vector (Wang and Chang 2003). The p3xARE/Luc vector, harboring three tandem repeats of ARE, was donated by X.L. Chen (Discovery Research, AtheroGenics Inc., Alpharetta, GA, USA) (Chen et al. 2003). The wild-type NRF2 expression vector (WT-NRF2) was a gift from H.S. So (Wonkwang University School of Medicine, Korea) (So et al. 2006). *NRF2* cDNA was subcloned into a pcDNA3.1(+) vector (Invitrogen, San Diego, CA, USA). For the transfection of reporter plasmids, we seeded HaCaT cells into six-well plates at a density of 80% the previous day. Cells were then transfected with a total of each luciferase reporter construct (2.5 μg) using LipofectAMINE plus (Invitrogen). To control for the efficiency of transfection, Renilla luciferase gene expression was monitored using either the pRL-CMV or pRL-TK vectors (Promega, Madison, WI, USA). For overexpression of WT-NRF2, we normalized the total plasmid concentration using the pcDNA3.1(+) empty vector. Thirty-six hours after transfection, the medium was replaced with fresh medium containing either vehicle (PBS) or iAs for 6 hr. After iAs exposure, we harvested cells and analyzed them for luciferase activity using a Dual-Luciferase Reporter Assay System (Promega).

For the investigation of the role of NRF2 in regulating *K16* gene expression, transfection of an NRF2 expression plasmid into HaCaT cells was carried out using LipofectAMINE 2000 (Invitrogen). Cells were cultured in 100-mm plates 24 hr before transfection. The expression plasmid WT-NRF2 (15 μg) was then transfected into the cells for 48–60 hr. As a negative control, we used 15 μg of the pcDNA3.1(+) empty vector.

### Electrophoretic mobility-shift assays (EMSA)

We extracted and measured nuclear proteins as described above. Nuclear protein/ DNA complexes were subjected to electrophoresis in nondenaturing 5% polyacrylamide gels containing 2% glycerol in 0.25% Tris-borate/EDTA buffer and transferred to Hybond-N^+^ nylon transfer membranes (Amersham Biosciences) for detection using the Light-Shift EMSA kit (Pierce) according to the manufacturer’s protocol, with minor modifications. We incubated 10-μg aliquots of nuclear extract with the DNA probe in a binding reaction buffer containing 10 mM Tris/HCl (pH 7.6), 50 mM KCl, 0.5 mM dithiothreitol, 0.25 mM EDTA, 5% glycerol, 2.5 mM MgCl_2_, 0.05% NP-40 detergent, and 2 μg of poly(dI-dC)·poly(dI-dC) for 30 min at room temperature. For supershift assays, 2 μg of either a polyclonal anti-NRF2 or an anti-c-Jun antibody (Santa Cruz Biotechnology) was added with the nuclear protein for 2 hr at 4°C before the labeled oligonucleotide probe was added. Biotin-labeled, double-stranded oligonucleotides WT-K16ARE (–157/–132, 5′-GGGGAACCTGGAGTCAGCAGT-TAGGA-3′), containing an ARE site (–148/ –140, underlined) in the human *K16* promoter region, and Mut-K16ARE (5′-GGGGAA-CCTGGAGTCAaaAGTTAGGA-3′, mutated GC box in the ARE) were prepared by Fasmac (Kanagawa, Japan). A consensus ARE probe was purchased from Panomics, Inc. (Redwood City, CA, USA). For competition binding of the K16 ARE-complexes, we used an unlabeled AP-1 consensus oligonucleotide (5′-TATC-GATAAGCTATGAGTCATCCGGG-3′). The binding specificity was confirmed in each case by the addition of a 100-fold molar excess of unlabeled oligonucleotide.

### CHX chase experiment

We investigated the posttranscriptional regulation of both the steady-state levels and half-life of the NRF2 protein by CHX chase analysis. Cells were incubated in serum-free medium in the absence or presence of iAs for 6 hr. The culture medium was then replaced with serum-free medium containing CHX (100 μg/mL). We prepared cell lysates at 0, 10, 30, 60, 120, and 240 min after iAs treatment. Whole-cell lysates were resolved by SDS-PAGE and immunoblotted with antibodies against NRF2.

### Statistics

All the data generated from at least three independent experiments and expressed as the mean ± SD were analyzed by the Student’s *t*-test. Statistical comparisons were made by logarithmic transformation of the normalized values. We considered *p*-values < 0.01 to be statistically significant.

## Results

### K16 expression is induced by iAs in HaCaT cells

**Figure 1:**
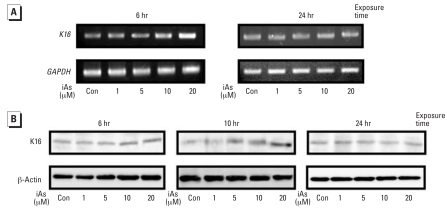
Dose-dependent effects of iAs on the expression of *K16* in HaCaT cells maintained in serum-free medium for 24 hr and then treated with iAs at various concentrations for 6 or 24 hr (*A*) or 6, 10, or 24 hr (*B*). Con, control. (*A*) After iAs treatment, total RNA was extracted from the cells, and 1-μg aliquots were amplified by semiquantitative RT-PCR using *K16* primers; *GAPDH* was amplified as an internal control. (*B*) Equal amounts of total protein extracts from the cells were subjected to Western blot analysis for K16; β-actin expression was analyzed as a loading control.

We wanted to determine whether the K16 mRNA is transcriptionally regulated by iAs, and treated HaCaT cells with this compound for various time periods over a range of doses. After treatment of HaCaT cells with 1–20 μM iAs, the expression of K16 mRNA was increased compared with the control at 6 hr (Figure 1A) but had declined to basal levels at 24 hr. The increase in the K16 protein levels after 6 hr of iAs exposure was just detectable at 10–20 μM, but a dose-dependent increase was more evident at 10 hr (Figure 1B). This enhancement of K16 expression had declined to basal levels at 24 hr.

### Identification of the iAs responsive region in the K16 gene promoter

**Figure 2:**
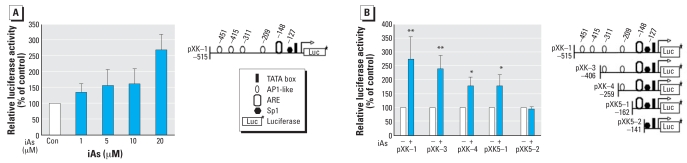
Response of the *K16* regulatory region to iAs using a luciferase reporter gene assay, with relative luciferase activities normalized for pRL-CMV activity and calculated as a percentage of the promoter activity in HaCaT cells without iAs exposure (control). Abbreviations: +, presence; –, absence; Con, control. (*A*) Transcriptional activation of *K16* gene by iAs (left) and the luciferase reporter gene construct harboring the –515-bp *K16* gene promoter region (pXK-1; right). (*B*) Luciferase activity of the iAs responsive region in the *K16* gene promoter in cells cotransfected with individual reporter constructs harboring various deletions of the 5′-flanking region of the *K16* gene promoter (left) and normalized for pRL-CMV, and the luciferase reporter gene constructs for individual promoter regions (right). See “Materials and Methods” for details of experiments. The values shown indicate the ratio of the luciferase activities in untreated cells and in cells treated with 20 μM iAs for 6 hr; luciferase activity with no treatment was defined as 100%. Values represent the mean ± SD of five to eight independent experiments, each run in triplicate. Each *p*-value was obtained by *t*-test following logarithmic transformation of normalized values. *p < 0.01, and **p < 0.001 compared with the untreated group.

To investigate the mechanisms underlying the transactivation of the *K16* gene by iAs, we first examined the response of the *K16* regulatory region to this compound using a luciferase reporter gene assay. The dose-dependent activation of *K16* transcription after iAs treatment was observed with a construct containing a 515-bp fragment of the *K16* promoter (Figure 2A). To further elucidate the region containing the iAs responsive element, we examined a series of deletions of the 5′-flanking region of *K16* gene. The ARE sequence in the pXK-5–1 vector contains an activator protein-1 (AP-1)–like element followed by a GC box. As shown in Figure 2B, an enhancement in the reporter activity levels was observed for the promoter constructs, pXK-1, 3, 4, and 5-1, in response to 20 μM iAs. A decline in reporter activity, however, depended on the number of AP-1–like sites, and the results for the pXK-5-1 construct show also that ARE is activated by iAs. In contrast, no significant activation was observed using a pXK-5-2 construct in response to 20 μM iAs.

### Expression of AP-1 transcriptions factor and c-Jun production following iAs treatment

**Figure 3:**
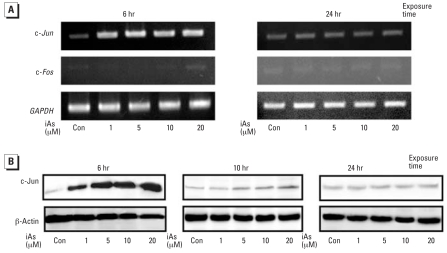
Effects of iAs on the expression of AP-1 transcription factors in HaCaT cells maintained in serum-free medium for 24 hr and then treated with iAs. Con, control. (*A*) Expression of c-*Jun*, c-*Fos*, and *GAPDH* genes examined by semiquantitative RT-PCR at 6 and 24 hr; *GAPDH* gene expression was analyzed as a loading control. (*B*) Western blot analysis of nuclear extracts from cells 6, 10, or 24 hr after iAs treatment using c-Jun and β-actin antibodies. Equal protein loading was confirmed by β-actin expression. Results were obtained from three independent experiments.

We examined AP-1 transcription factors c-*Jun* and c-*Fos* expression in iAs-treated HaCaT cells by semiquantitative RT-PCR. iAs-induced c-*Jun* expression was observed during the first 3 hr after treatment (data not shown). An appreciable induction of c-*Jun* was also confirmed after 6 hr, but this was down-regulated by 24 hr after iAs treatment (Figure 3A). In contrast, the expression of c-*Fos* was only transiently detectable at 3 hr (data not shown) but was not observed during the 6–24 hr period of this experiment. As shown in Figure 3B, iAs-enhanced c-Jun production can be observed in a dose-dependent manner at 6 hr, but it declines from 10 to 24 hr.

### iAs potently induces the translocation of NRF2 and activates the ARE of the K16 promoter

**Figure 4:**
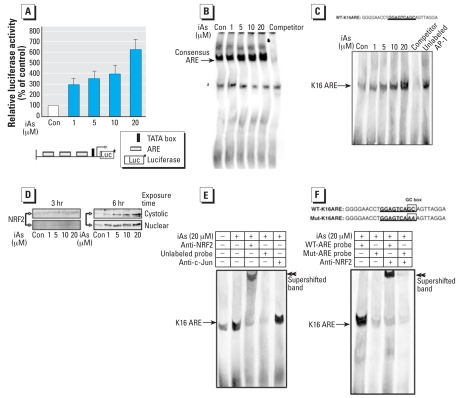
iAs potently stimulates the ARE and induces production and translocation of NRF2 into the nucleus of HaCaT cells. Abbreviations: +, presence; –, absence; Con, control. (*A*) Effect of iAs on ARE-driven promoter activity determined using cotransfection of the p3xARE/Luc reporter gene construct (shown schematically in the lower panel), containing three copies of the ARE, into HaCaT cells with the pRL-TK vector encoding Renilla luciferase. See “Materials and Methods” for details. Values shown indicate the ratio of the luciferase activities in untreated cells and in cells treated with 20 μM iAs for 6 hr; luciferase activity with no treatment was defined as 100%. Values represent the mean ± SD of three independent experiments, each run in triplicate. (*B*) Results of EMSA analyses performed using iAs-stimulated nuclear extracts and a biotinylated consensus ARE probe. The arrow indicates the consensus ARE probe-nuclear protein band shifts; an unlabeled consensus ARE probe was used as a competitor oligo. (*C*) EMSA of nuclear extracts prepared from cells treated with iAs for 6 hr using a biotinylated WT-K16ARE probe (5′-GGGGAACCTGGAGTCAGCAGTTAGGA-3′, with the wild-type ARE sequence found in the *K16* promoter from –157 to –132 bp underlined). An unlabeled WT-K16ARE probe was used as a competitor. To determine the binding specificity of ARE, an unlabeled AP-1 oligonucleotide was added to the iAs-treated nuclear extracts before the addition of the labeled WT-K16ARE probe. The arrow indicates shifted K16ARE-nuclear protein complexes. (*D*) Nuclear accumulation of NRF2 stimulated by iAs in HaCaT cells maintained in serum-free medium for 24 hr and then treated with iAs for 3 or 6 hr. Cytoplasmic and nuclear extracts were then prepared and subjected to Western blot analysis using polyclonal NRF2 antibodies; results shown represent three independent experiments. (*E*) Regulation of the ARE site within the *K16* gene promoter after iAs treatment. The WT-K16ARE probe was incubated with nuclear extracts prepared from cells cultured in the absence (–) or presence (+) of 20 μM iAs for 6 hr. For supershift EMSA analysis, the nuclear extracts were incubated with either an anti-NRF2 or anti-c-Jun antibody before the addition of labeled probe. The binding specificity was confirmed by the addition of excess unlabeled WT-K16ARE probe. The arrow indicates shifted complexes, and double arrowheads indicate supershifted K16ARE-NRF2 complexes. (*F*) In a similar experiment to the one shown in (*E*), either a WT-K16ARE or Mut-K16ARE probe was incubated with nuclear extracts of cells cultured in the presence of 20 μM iAs for 6 hr. The GC box of Mut-K16ARE was substituted with AA as indicated. Anti-NRF2 antibody was used in the supershift assays. The arrows and double arrowheads indicate shifted and supershifted complexes, respectively. ^***a***^Nonspecific band.

The results of our reporter assays suggested that iAs stimulates not only the AP-1–like sites but also the ARE site within the *K16* gene promoter in HaCaT cells (Figure 3). In addition, several oxidative stress agents and toxic chemicals, including iAs, have been reported to induce the expression of ARE-dependent genes in several cell types (Pi et al. 2003; Sakurai et al. 2005). On the basis of our observations and some recent reports, we thus hypothesized that iAs would have the ability to activate the ARE of the *K16* gene promoter directly, resulting in the induction of *K16* expression in HaCaT cells. To confirm that the K16 ARE indeed functions as an iAs-responsive transcriptional control element, we performed transient transfections of HaCaT cells with a p3xARE/Luc construct and then subjected these cells to iAs for 6 hr. As shown in Figure 4A, treatment of HaCaT cells with iAs results in a dramatic increase in ARE-driven promoter activity. Likewise, EMSA using a consensus ARE probe show that iAs-induced ARE-binding complexes increase markedly, in a dose-dependent fashion (Figure 4B). These results indicate that iAs has the ability to activate the ARE-driven genes. We performed further EMSA experiments using an ARE probe specific to the *K16* proximal promoter region (WT-K16ARE) and found that K16ARE–nuclear protein complexes formation is augmented by iAs in a dose-dependent manner (Figure 4C). Moreover, the formation of these complexes is specifically inhibited by the addition of excess unlabeled oligonucleotide competitor (Figure 4B,C), whereas an excess of an unlabeled AP-1 probe competes only marginally for K16ARE binding (Figure 4C).

The NRF2 transcription factor has been shown to bind to AREs upon translocation into the nucleus, resulting in the induction of ARE-mediated genes (Wakabayashi et al. 2004). To examine whether iAs induces and translocates NRF2 into the nucleus in HaCaT cells, we treated these cells with iAs for either 3 or 6 hr. As shown in Figure 4D, a dose-dependent accumulation of NRF2 protein was observed in the nucleus upon treatment with iAs for 6 hr. This was not observed in the parallel experiment performed over the 3-hr time course.

Supershift EMSA analysis using an NRF2 antibody showed that the iAs-induced and iAs-translocated NRF2 protein binds to the WT-K16ARE probe containing the ARE sequence of the *K16* proximal promoter region (5′-GGAGTCAGC-3′) that comprises an AP-1–like site and a GC box, whereas the supershift of c-Jun was not observed (Figure 4E). To identify whether the GC box is dispensable for the iAs-stimulated binding activity of NRF2, we next performed EMSA analyses with either WT- or a Mut-K16ARE probe containing an intact AP-1–like element but a mutated GC box. As shown in Figure 4F, the K16ARE–nuclear protein complexes and supershifted bands that were enhanced by iAs treatment were largely abolished by the addition of the Mut-K16ARE probe.

### iAs stabilizes the NRF2 protein

**Figure 5:**
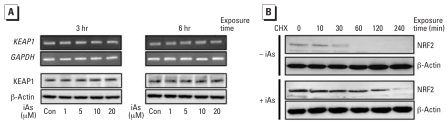
iAs enhances the stability of the NRF2 protein. Abbreviations: +, presence; –, absence; Con, control. (*A*) Expression levels of KEAP1 and GAPDH mRNA determined by semiquantitative RT-PCR in HaCaT cells cultured in serum-free medium for 24 hr and then treated with iAs for 3 or 6 hr (upper panels); cytoplasmic extracts from iAs-treated cells were subjected to Western blot analysis for KEAP1 and β-actin (lower panels). (*B*) Posttranscriptional regulation of both the steady-state level and half-life of NRF2 protein shown by Western blot analysis (see “Materials and Methods” for details). β-Actin was used as an internal control. Similar results were obtained from three independent experiments.

We examined the effects of iAs treatment on the function of KEAP1 in HaCaT cells. Treatment with iAs did not alter the expression levels of KEAP1 mRNA or protein over either a 3 or 6 hr time course (Figure 5A). Next, we examined the effects of iAs on the expression of NRF2 mRNA in HaCaT cells. Exposure to iAs did not significantly alter the steady-state levels of NRF2 mRNA (data not shown). Production of NRF2 protein, however, was observed to increase in both a dose-and time-dependent manner (Figure 4D). To further examine the stabilization of NRF2 protein by iAs, we monitored the decay of basal and iAs-induced NRF2 proteins after inhibition of protein synthesis by CHX (Figure 5B). The results of this analysis revealed that the NRF2 protein levels decrease by approximately 50% within 30 min of treatment with CHX in cells that had not been exposed to iAs. Only trace amounts of NRF2 are then detectable after 60 min of exposure to CHX in these cells. The HaCaT cells were then pretreated with iAs for 6 hr before their exposure to CHX in a similar timecourse experiment. The levels of NRF2 in these iAs-treated cells were again found to decrease by about 50%, but only after 120 min of CHX exposure.

### NRF2 plays a crucial role in the regulation of K16 gene expression in HaCaT cells

**Figure 6:**
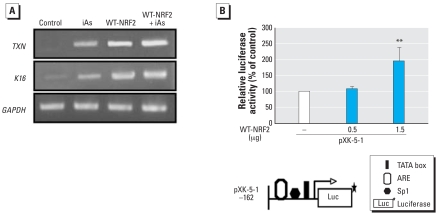
NRF2 directly induces the *K16* gene in HaCaT cells. Abbreviations: +, presence; –, absence; Con, control. (*A*) Cells were transiently transfected with empty vector pcDNA3.1 (control) or expression vector WT-NRF2 as described in “Materials and Methods,” and the expression of *TXN*, *K16*, and *GAPDH* was examined by semiquantitative RT-PCR using *GAPDH* expression levels as an internal control. (*B*) Cells were transfected with WT-NRF2, together with the pXK-5–1 and the Renilla luciferase vector pRL-TK (the pXK-5-1 luciferase vector is shown schematically in the lower panel). Transfected cells were incubated for 48 hr and then analyzed for luciferase activity as described in “Materials and Methods.” Luciferase activity in cells transfected with pcDNA3.1 was set as 100%. The values represent the mean ± SD of four independent transfection experiments, each run in triplicate. We obtained *p*-values by *t*-tests following logarithmic transformation of normalized values. **p < 0.01 compared with the control group (pcDNA3.1).

To confirm the functional role of NRF2 in the induction of *K16* gene expression by iAs, we investigated whether the expression of K16 mRNA is induced by the overexpression of NRF2 (WT-NRF2) in HaCaT cells. We also investigated the expression of the detoxification gene *TXN*, which is highly induced by a variety of oxidative stimuli through NRF2-mediated ARE transactivation (Kim et al. 2001). The expression of *TXN* gene in untransfected cells after treatment with iAs was stronger than that of the control cells (Figure 6A). When the cells were transfected with WT-NRF2 and then treated with or without iAs, the expression of TXN mRNA was augmented markedly compared with the empty-vector control. Similarly, the expression of K16 mRNA was also induced in cells transfected with WT-NRF2 in the absence or presence of iAs. We next performed a transient transfection of HaCaT cells with the pXK-5–1 luciferase vector together with the WT-NRF2 vector. The overexpression of NRF2 in increasing concentrations resulted in significant enhancement of the ARE-mediated *K16* promoter activation (Figure 6B).

## Discussion

In the present study, we showed for the first time that iAs induces the transcriptional activation of *K16* in the human keratinocyte cell line, HaCaT, through the ARE present in its gene promoter. It has been reported previously that treatment with iAs enhances the production and translocation of NRF2 into the nucleus in several cell types. However, until now it has remained uncertain whether the induction of NRF2 by iAs mediates the transcriptional activation of the *K16* gene in keratinocytes. In our current experiments, we demonstrated that iAs elongates the half-life of the NRF2 protein, which results in its increased expression levels. Furthermore, this iAs-induced NRF2 protein was shown to bind to the ARE sequences in the promoter region of the *K16* gene. Finally, by overexpressing NRF2, we have clarified that its induction is involved in not only the gene expression of the detoxification gene *TXN*, but also in the upregulation of *K16* expression in HaCaT cells through the ARE in the *K16* gene promoter. These experiments indicate an important and novel function for NRF2 in the regulation of *K16* in keratinocytes and also help to further explain the molecular mechanisms underlying arsenic-mediated epidermal hyperkeratosis.

In our present experiments, the expression levels of K16 mRNA and protein were indeed found to be enhanced by iAs in a dose-dependent manner (Figure 1). In addition, luciferase assays of the *K16* promoter revealed that iAs enhances its activity in a dose-dependent fashion, which is stimulated by AP-1–like sites and an ARE (Figure 2). The promoter of the human *K16* gene was recently cloned and sequenced, and several AP-1–like sites were found within the –515-bp region of the gene (Wang and Chang 2003). AP-1 transcription factor can be formed by the dimerization of either Jun or Jun/Fos family members (Eferl and Wagner 2003). In the present study, the increased expression of c-*Jun*, but not c-*Fos* was evident in the nuclei of HaCaT cells after iAs treatment (Figure 3). Our findings thus suggest that the activation of c-Jun/AP-1 is one of the essential steps in the regulation of *K16* gene expression by iAs exposure in HaCaT cells.

It has been well documented that the ARE core sequence includes an AP-1–like binding site (TGAC/GTCA), followed by a GC box (Rushmore et al. 1991; Xie et al. 1995). We have found in our current analyses that the AP-1–like site within the *K16* promoter region from –148 to –140 bp (5′-GGAGTCAGC-3′) resembles a consensus ARE sequence. Recent studies have shown that AREs can be specifically bound by complexes of several basic-leucine zipper transcription factors, including NRF2 (Ishii et al. 2000; Moi et al. 1994). NRF2 heterodimerizes with either AP-1 or small MAF (MAFG, MAFK, and MAFF) proteins (MAF, v-maf musculoaponeurotic fibrosarcoma oncogene homolog) and binds to the ARE to induce the transcription of ARE-mediated genes (Motohashi et al. 2002). In the present investigation, EMSA and supershift assays showed that the NRF2 proteins in the nuclei bind to the ARE sequences of *K16* promoter region after iAs exposure. iAs-induced c-Jun, however, does not bind to this ARE (Figure 4E). c-Jun may thus act on other AP-1 sites within the *K16* promoter region. These results also suggest that other heterodimer partners of NRF2 are involved in the ARE regulation of *K16* promoter region underlying iAs-mediated the *K16* gene expression. Gel shifts with an K16Mut-ARE probe (harboring a mutation in the ARE GC box) clearly show that the ARE sequence in the *K16* promoter, particularly the terminal GC dinucleotide, is essential for mediating iAs-induced *K16* transactivation and NRF2 binding (Figure 4F). Several investigations have suggested that the GC nucleotides within the ARE are essential for both the basal and oxidative stress–induced activities of the ARE-related genes, NAD(P)H dehydrogenase quinone 1 (*NQO1*) and glutamate-cysteine ligase catalytic subunit (*GCLC*) (Wasserman and Fahl 1997; Wild et al. 1998). Our current results are consistent with these earlier studies in showing that the formation of the iAs-responsive NRF2/ARE complexes is reduced by a mutation in the GC box. Collectively, our present observations reveal a new molecular mechanism in which iAs-induced *K16* gene expression is also regulated by activation NRF2/ARE pathways.

It has been widely accepted that oxidative stress disrupts sequestration of NRF2 by KEAP1, permits NRF2 translocation to the nucleus, and transactivates the expression of various NRF2-mediated genes (Dinkova-Kostova et al. 2002; McMahon et al. 2003). Our present study showed that iAs elongates the half-life of the NRF2 protein but has no effects upon KEAP1 expression (Figure 5). Other studies have also demonstrated that the production of NRF2 is increased by various inducers via posttranscriptional control (Nguyen et al. 2003; Stewart et al. 2003). Several earlier reports also indicated that either oxidative stress or antioxidant substances stabilize the expression of the NRF2 protein, either by directly modifying the cysteine residues on KEAP1 to disrupt the NRF2/KEAP1 complex (Dinkova-Kostova et al. 2002) or by facilitating the release of NRF2 through the phosphorylation of the NRF2/KEAP1 complex (Bloom and Jaiswal 2003). These findings are largely consistent with our present finding that iAs stabilizes the expression of NRF2 in HaCaT cells by elongating the protein half-life.

Recently, Wakabayashi et al. (2003) demonstrated that NRF2 accumulates in the nucleus at constitutively high levels and produces various cytoprotective genes in embryonic fibroblast- and liver-derived *Keap1*-null mice. Surprisingly, these *Keap1*-deficient mice also show a thicker stratum corneum epidermis, abnormal keratinization, and cornification in the esophagus and forestomach (hyperkeratosis). *K6* was found to be strongly expressed in the esophageal epithelium of these mice. These results indicate that *K6* is also a target gene of NRF2. In addition, the promoter of the *K6* gene bears a remarkable sequence similarity to the *K16* promoter (Jiang et al. 1993). Therefore, we examined whether *K16* gene expression is also regulated by NRF2. In the present study, the gene expression and transactivation of *K16* were dramatically induced by transfection with WT-NRF2 via in HaCaT cells, clearly demonstrating that NRF2 acts as a direct transcriptional regulator of the *K16* gene (Figure 6). In addition, we also showed that transfection of HaCaT cells with WT-NRF2 induces the expression of detoxification gene *TXN* (Figure 6A). NRF2 may thus have a major role to play in the development of hyperkeratosis, whereas the expression and induction of NRF2 is implicated in cell protection against a variety of genotoxic and cytotoxic effects. Hence, based on these results and on the findings from studies of *Keap1* knockout mice, iAs may both cause hyperkeratosis and induce detoxification enzymes via the modification of NRF2. Given that there are both beneficial and adverse effects of NRF2 activity, caution will therefore be needed when using antioxidants for prevention and therapy. Although further investigations are needed, we believe that our findings provide important clues for the design of future therapies for arsenic-mediated hyperkeratosis and for treatments involving the molecular targeting of NRF2.

## Conclusion

Our findings clearly demonstrate that the induction of the *K16* gene in human keratinocytes by iAs depends on NRF2 activation. Our results thus represent a valuable initial effort to elucidate the relationship between the *K16* gene and the NRF2 transcription factor, which may be responsible for hyperkeratosis.
